# Feasibility of Cognitive Functions Screened With the Montreal Cognitive Assessment in Determining ADL Dependence Early After Stroke

**DOI:** 10.3389/fneur.2018.00705

**Published:** 2018-08-27

**Authors:** Tamar Abzhandadze, Lena Rafsten, Åsa Lundgren-Nilsson, Katharina S. Sunnerhagen

**Affiliations:** ^1^Institute of Neuroscience and Physiology, Rehabilitation medicine, University of Gothenburg, Gothenburg, Sweden; ^2^Department of Occupational Therapy and Physiotherapy, Sahlgrenska University Hospital, Gothenburg, Sweden; ^3^Centre for Person-Centred Care (GPCC), University of Gothenburg, Gothenburg, Sweden

**Keywords:** stroke, rehabilitation, cognition, activities of daily living, stroke outcome, assessments, acute stroke

## Abstract

**Objective:** To investigate the feasibility of assessing cognitive function using the Montreal Cognitive Assessment (MoCA) given 36–48 h post stroke to explain dependence in activities of daily living (ADL).

**Methods:** This is a cross-sectional, exploratory study. Cognitive function and basic ADL were assessed with the MoCA and the Barthel Index (BI), respectively, within 36–48 h of admission. Neurological functions were assessed with the National Institute of Health Stroke Scale (NIHSS) upon admittance to the hospital. Binary logistic regression analyses were performed to assess the feasibility of the MoCA in explaining ADL dependence.

**Results:** Data were available for 550 patients (42% females, mean age 69 years). Moderate correlations (*r*_*s*_ > +0.30, *p* < 0.001) were found between the total score on the BI, MoCA, and visuospatial/executive functions. The regression analysis model including only MoCA as an independent variable had a high sensitivity for explaining ADL dependence. However, the model with independent variables of MoCA, NIHSS, and age had the best area under the curve value (0.74).

**Conclusions:** Cognitive functions assessed with the MoCA partly explain ADL dependence 36–48 h post stroke. Stroke-related neurological deficits and age should be additional considerations.

## Introduction

Cognitive functions play an important role in patients' rehabilitation setting management (1) and safe discharge. Few studies have investigated the utility of assessing patients' cognitive functions during the early stages of stroke onset to explain activity-related outcomes. As even mild stroke can lead to cognitive impairments and influence patients' everyday functioning (2), it is important to identify these difficulties.

The Montreal Cognitive Assessment (MoCA) is a recommended tool for assessing cognitive functions in patients with acute stroke (3, 4). Good validity and reliability were reported for those with mild to moderate stroke (4). Studies performed on a subacute stroke population showed a positive association between impaired cognitive function assessed with the MoCA and a high level of global disability (5). Poor executive and memory functions were positively associated with dependence in activities of daily living (ADL) (6). However, explanatory factors for favorable ADL outcomes 3 to 12 month post stroke were stroke localization (7), younger age, less severity of neurological deficits, and good function in the upper extremities (7, 8).

The length of stay after the stroke has decreased substantially and particularly, the patients with very mild to mild neurological deficits, face very short hospital stay (9). The clinicians often have only couple of days to identify stroke related difficulties. Thus, there is increased need of very early assessments of cognitive functions. The MoCA and basic ADL with the Barthel Index (BI) are both commonly used instruments for this, in order to plan discharge. Whether cognitive functions assessed by the MoCA can explain patients' ADL ability at the early stage of stroke onset remains unknown. The aim of this study was therefore to investigate the feasibility of the MoCA to explain ADL dependence 36–48 h post stroke.

## Materials and methods

### Study design and participants

This was a cross-sectional, exploratory study. The study population consisted of stroke patients screened for the Gothenburg Very Early Supported Discharge Study (GOTVED) (10). The participants for GOTVED were enrolled at the acute stroke care unit in Gothenburg, Sweden, between May 2011 and April 2016. The inclusion criteria for this study were stroke diagnosis, complete data on the BI and the MoCA. The exclusion criteria were patients with Amaurosis fugax, subarachnoid hemorrhage and Transient Ischemic Attack (TIA). The Declaration of Helsinki was followed. The regional ethical review board in Gothenburg approved the study (042–11, T 392-17).

### Procedure

All patients admitted to the acute stroke care unit were screened within 36–48 h of admission by clinical occupational therapists who administered the MoCA and the BI. Information about patients' stroke-related neurological deficits upon admittance to the hospital, independence in ADL, and mobility prior to the stroke, stroke localization, and paresis in the dominant/non-dominant upper extremity was obtained from the medical charts. The stroke was classified according to Oxfordshire Community Stroke Project Classification (OCSP) (11).

### Assessments

#### Dependent variable

The patients' independence in basic ADL was assessed with the BI (12). The total score range on the BI is 0 to 100, with higher scores indicating higher level of ADL independence.

#### Independent variables

Patients' sex, age, length of stay at an acute stroke care unit, stroke localization, independence in ADL, and mobility prior stroke, and paresis in the dominant/non-dominant upper extremity were used in the study. Cognitive functions were assessed with the MoCA (3). The score range on the MoCA is 0 to 30; the cutoff value of ≥26 indicates normal cognitive functioning (13). MoCA evaluates cognitive domains in visuospatial/executive functions, attention, language, abstraction, delayed reproduction, and orientation (3). Stroke-related neurological deficits were assessed with the NIHSS (14). The total score range is 0 to 42, with higher scores indicating more severe neurological impairments. Cognitive function was estimated with Cog-4 (15), which is based on four items from the NIHSS; awareness, executive functions, language, and inattention. The range of scores is 0 to 9 points, and “0” indicates no cognitive deficits.

### Statistical analysis

Categorical variables are presented as numbers and percentages; continuous variables are shown as mean, median, standard deviation (SD), and range. Confidence intervals (95% CI) are given when appropriate. For descriptive purpose only, the MoCA scores were dichotomized (≤ 25 cognitive impairments and ≥ 26 normal cognitive functions) (13).

Many variables had skewed distributions; therefore, non-parametric statistical tests were used. Differences between groups were studied with Pearson's χ^2^ test for dichotomous variables and Mann–Whitney *U-*test for continues variables. Correlations between the BI and the independent variables were analyzed with Spearman's rank order correlations test (r_s_). The correlation values were interpreted as small (*r* < ±0.29) or medium (*r* = ±0.30 to ±0.49) (16).

**Figure 1:**
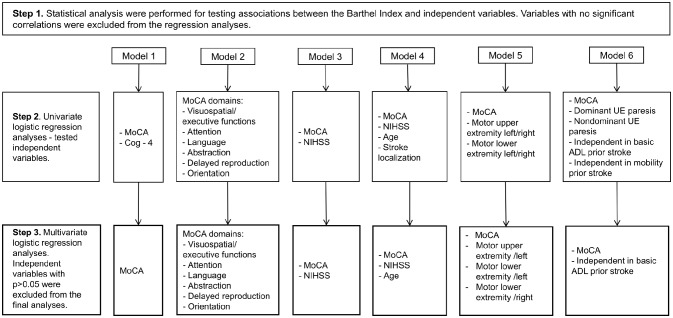
The procedure for creating six logistic analysis models (outcome ADL-dependence, the Barthel Index <95). MoCA, The Montreal Cognitive Assessment; NIHSS, the National Institute of Health Stroke Scale. UE, upper extremity.

Binary logistic regression analyses were performed to assess the associations between MoCA and the BI. Regarding dependent variables, the BI was dichotomized and coded as “dependent-1” (<95) and “independent-0” (>95) (17, 18). The independent variables were chosen according to previous literature (6–8) and clinical experience. Age, the MoCA and the NIHSS scores were included as continuous variables in the models. The MoCA and the NIHSS items were categorical variables. The process of independent variable selection and regression analysis model building was performed in three steps (Figure 1). Area under the curve (AUC) was investigated to study the fit of the models. Results were interpreted as follows: 0.7–0.9 as moderate accuracy, and 0.5–0.7 as low accuracy (19). A separate analysis was done for the MoCA adjusted to language difficulties (the NIHSS item-language).

All tests were 2-tailed with 5% significance. Type I error was controlled with the Bonferroni correction test. Data were analyzed using SPSS (version 22, SPSS, Inc., Chicago, IL, USA).

## Results

**Figure 2:**
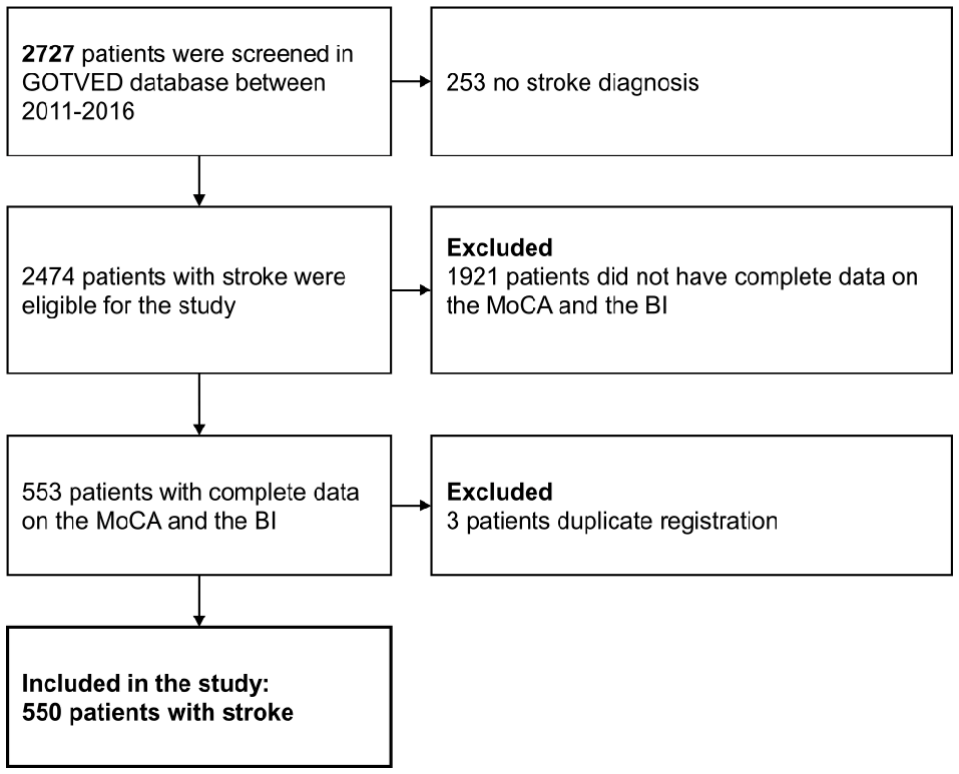
Flowchart of the participants. GOTVED, the Gothenburg Very Early Supported Discharge Study, MoCA, the Montreal Cognitive Assessment; BI, the Barthel Index.

In total 2727 patients were screened for the GOTVED study between 2011 and 2016. Of those patients 2474 had stroke diagnosis. The complete data on the BI and the MoCA were present on 553 patients. Older patients (*p* < 0.001) had missing data on both assessment instruments, regarding the sex no significant differences were found. Furthermore, three patients had duplicate registration. Five-hundred and fifty patients were included in the study (Figure 2).

### Characteristics of the study population

Of the 550 included patients, 317 (58 %) were male, and 323 (59%) scored < 26 on the MoCA. A majority of the patients (*n* = 367, 67%) had NIHSS 0–2. Patients who were dependent in ADL (BI <95) were more women, were also significantly older and had impaired cognitive functions, with a median MoCA score of 22. Five-hundred and thirty-four (97%) patients and 546 (99%) patients were independent in basic ADL and mobility prior to stroke, respectively (Table 1).

**Table 1:** Characteristics of the study population^*^.

**Variables**	**All patients**	**BI < 95**	**BI ≥ 95**	***P*-value^¶^**
	***N* = 550**	***N* = 227**	***N* = 323**	
Female sex, *n* (%)	233 (42)	106 (47)	126 (39)	<0.001^§^
Age, y, mean (*SD*)	69 (15)	74 (13)	66 (15)	<0.001^†^
Length of hospital stay, median (range)	6 (2–43)	9 (2–43)	5 (2–36)	
BI, median (range)	95 (10–100)	75 (10–90)	100 (95–100)	
MoCA, median (range)	25 (3–30)	22 (4–30)	26 (3–30)	<0.001^†^
Cog – 4, median (range)	0 (0-7)	0 (0–7)	0 (0–7)	<0.05^†^
NIHSS, median (range)	1 (0–22)	2 (0–22)	1 (0–14)	<0.001^†^
Dominant UE paresis, *n* (%)	168 (30)	68 (30)	100 (31)	<0.01^§^
Nondominant UE paresis, *n* (%)	227 (41)	108 (48)	119 (37)	<0.01^§^
Independent in basic ADL prior stroke, *n* (%)	534 (97)	214 (40)	320 (60)	<0.001^§^
Independent in mobility prior stroke, *n* (%)	446 (99)	222 (41)	321 (59)	<0.001^§^
Total anterior circulation stroke, *n* (%)	11 (2)	8 (3)	3 (1)	
Partial anterior circulation stroke, *n* (%)	77 (14)	39 (17)	38 (12)	
Posterior circulation syndrome, *n* (%)	187 (34)	87 (38)	100 (31)	
Lacunar syndrome, *n* (%)	232 (42)	77 (34)	155 (48)	
Intracerebral hemorrhage, *n* (%)	43 (8)	16 (7)	26 (8)	

* *The sum may vary due to missing data*.

¶ Statistical difference between groups with BI < 95 versus BI > 95

† Mann–Whitney U-test, two tailed,

§ *Pearson's chi-squared test, two tailed*.

*UE, upper extremity; BI, the Bathel Index; MoCA, the Montreal Cognitive Assessment; NIHSS, the National Institutes of Health Stroke Scale; Cog-4, the sum of four items from the NIHSS*.

### Correlations with the ADL performance

The total BI score was associated with patients' demographic characteristics and stroke-related neurological and cognitive deficits (Table 2). Medium, positive correlations (*r*_*s*_ > +0.30, *p* < 0.001) were found between the BI, total MoCA score, and the MoCA cognitive domain-visuospatial/executive functions. Small, positive correlations (*r*_*s*_ < +0.30, *p* < 0.001 to *p* < 0.05) were found between the BI and the MoCA cognitive domains attention, language, abstraction, delayed reproduction and orientation. Small, negative correlations (*r*_*s*_ < −0.30, *p* < 0.001 to *p* < 0.05) were identified between the BI, age, sex, NIHSS, Cog-4, and the NIHSS item paresis in left/right upper extremity (Table 2).

**Table 2:** Correlations between the Barthel Index^*^, demographic features, stroke-related neurological, and cognitive functions, and patients' functioning prior to stroke.

**Independent variables**	**r_s_**	***P*-value**
Age	−0.28	<0.001
Sex^†^	−0.10	0.022
NIHSS^‡^	−0.27	<0.001
COG-4^§^	−0.12	0.006
Visuospatial/executive functions	0.35	<0.001
Attention	0.24	<0.001
Language	0.16	<0.001
Abstraction	0.15	<0.001
Delayed reproduction	0.25	<0.001
Orientation	0.18	<0.001
MoCA^¶^ – total score	0.36	<0.001
Paresis of the right upper extremity	−0.10	0.024
Paresis of the left upper extremity^**^	−0.20	<0.001

*r_s_, Spearman's rank order correlations test*.

* *Barthel Index is scored from 0 to 100, where 100 indicates independence in activities of daily living*.

† *Sex-1 = man, 2 = women*.

‡ *NIHSS, the National Institute of Health Stroke Scale scores range from 0 to 42, where 0 indicates no neurological disabilities*.

§ *Cog−4, the sum of four items from the NIHSS, ranging from 0 to 9, where 0 indicates no cognitive deficits*.

¶ *MoCA, Montreal Cognitive Assessment, scores range from 0 to 30, where 30 indicates no cognitive deficits*.

** *Paresis in right/left upper extremity – scores range from 0 to 4, where 0 indicates no motor deficits*.

### Associations of the MoCA with the ADL performance

Six independent models were created based on the independent variables patients' age, MoCA total score, each cognitive domain of the MoCA, the NIHSS total score, four items from the NIHSS: motor upper extremity left/right and motor lower extremity left/right. The model (#1) including only the MoCA had the best sensitivity, showing that normal cognitive functions decreased the odds of being ADL dependent. The MoCA adjusted for language impairments (*p* < 0.001, OR = 85, 95 % CI 0.81–0.89, AUC = 0. 69) showed somewhat decreased sensitivity (39.6) and increased specificity (86.0) for ADL dependence. The model (#4) including age, total MoCA score, and total NIHSS score had the highest association value with ADL dependence (AUC = 0.74), indicating that younger age, normal cognitive functions, and less stroke severity decreased the odds of ADL dependence (Table 3).

**Table 3:** Independent variables associated with ADL dependence measured with the BI.

RAM^*^	**Independent variables**	**Adjusted**	**Adjusted OR**	**AUC**	**Sensitivity**	**Specificity**
		***P*-Value**	**(95% CI)**			
1	MoCA	<0.001	0.86 (0.82–0.90)	0.69	87.3	36.6
2	Visuospatial and executive functions	<0.001	0.70 (0.60–0.82)	0.71	82.9	46.7
	Attention	0.073	0.85 (0.72–1.01)			
	Language	0.177	1.16 (0.94–1.43)			
	Abstraction	0.662	0.93 (0.69–1.26)			
	Delayed reproduction	0.001	0.80 (0.70–0.91)			
	Orientation	0.702	0.95 (0.74–1.23)			
3	MoCA	<0.001	0.87 (0.83–0.91)	0.72	83.9	47.7
	NIHSS	<0.001	1.18 (1.10–1.26)			
4	MoCA	<0.001	0.89 (0.85–0.94)	0.74	81.0	51.8
	NIHSS	<0.001	1.18 (1.10–1.27)			
	Age	<0.001	1.03 (1.01–1.04)			
5	MoCA	<0.001	0.86 (0.82–0.89)	0.72	50.5	84.2
	Motor UE/left	0.05	1.53 (1.00–2.34)			
	Motor LE/right	<0.01	1.80 (1.20–2.69)			
	Motor LE/left	0.33	1.31 (0.76–2.25)			
6	MoCA	<0.001	0.87 (0.83–0.90)	0.70	87.0	38.3
	ADL ability prior stroke	0.023	5.98 (1.27–28.1)			

* *Regression analyses models. OR, odds ratio; AUC, area under curve; UE, upper extremity, LE, lower extremity. BI, Barthel Index (1 [<95] dependent, 0 [>95] independent); MoCA, Montreal Cognitive Assessment (0 severe cognitive impairments, 30 no cognitive impairments); NIHSS, National Institute of Health Stroke Scale (0 no symptoms, 42 severe neurological impairments); ADL, activities of daily living. Motor UE/left and motor LE left/right, 0 no drift, 4 no movement. Adjusted OR for explaining ADL dependence in the BI is associated with a one-unit decrease in the MoCA, visuospatial/executive functions, and delayed reproduction, and a one-unit increase in NIHSS, motor UE/left and motor LE/right*.

## Discussion

The study findings suggest that cognitive functions measured with the MoCA administered 36–48 h post stroke can reflect on ADL performance. Furthermore, patients with impaired cognitive functions, older age, and severe neurological deficits at this time are more likely to be dependent. The present findings strengthen the recommendation for using the MoCA in acute stroke settings to identify cognitive changes (20, 21). Additionally, the results provide more knowledge about the possible use of the MoCA as one of the variables associated with ADL dependence.

When and what kind of assessments should be performed at acute stroke care units has been argued. Stroke recovery is a dynamic process, and much occurs during the first days. Therefore, screening patients for cognitive functions is sometimes not prioritized during the early stages of stroke onset and practitioners would rather do ADL assessments than cognitive screening. However, in patients with mild to moderate stroke, it is possible that there is a “cognitive reserve” while performing basic ADL, since they are well-established, automatized activities. Thus, post stroke cognitive changes can be hard to identify based only on basic ADL assessments. According to Crichton et al. (22) even patients with mild stroke may experience cognitive deficits, leading to activity and participation limitations. In this group of patients, early cognitive screenings could be necessary due to short hospital stays (9).

Based on our study results, assessing cognitive functions with the MoCA could be informative (21) for understanding ADL dependence even if MoCA has showed low level of association. There are several factors, which could affect ADL performance at very early stage of stroke onset. Regression analyses model including MoCA and ADL ability prior stroke has showed high sensitivity but relatively low specificity. This indicates that premorbid factors might influence ADL performance early after stroke. According to Almenkerk et al. (23) comorbidities such as diabetes mellitus, history of stroke as well as cognitive functioning prior stroke and living status could also influence the outcomes.

In this study, 59% of the patients had MoCA scores under the threshold for normal cognitive functioning. The MoCA has high sensitivity for cognitive changes (13, 24); however, several studies mention that the cutoff value of ≥ 26 is too high for stroke populations (24, 25). Thus, it is possible that due to this high threshold, MoCA identified more patients with possible cognitive deficits (21). However, it is unclear if deficits were valid or could be caused by other post-stoke conditions; Even though MoCA has shown to be feasible in acute stroke, (20) it is possible that it has identified cognitive changes related to post stroke fatigue (26), delirium (27), or emotional reactions (28) which are common after stroke. Furthermore, patients in our study in whom the MoCA identified impaired visuospatial/executive functions and delayed reproduction were more likely to be dependent in ADL. However, regression analyses model including MoCA's cognitive domains showed low sensitivity and specificity. The results are partially in line with other studies showing that cognitive domains like attention, language (29), executive functions, and delayed reproduction (6) might have an impact on ADLs, highlighting executive functions as the most important. Decline in executive and memory functions is usually related to age (30). Thus, based on these study results, the MoCA total score could be recommended, rather than a score on a particular cognitive domain, to explain ADL dependence.

The NIHSS and the MoCA were pooled in the analysis testing whether cognitive functions remained significantly associated with ADL dependence. The AUC value and specificity of the model increased, but precession of MoCA slightly worsened compared with the first model. Applicability of the MoCA depends on the type and severity of neurological deficits. The MoCA is difficult to administer to patients with severe stroke (21). Thus, for these patients the NIHSS could be an alternative for explaining ADL dependence (21). In a study by Kwakkel et al. (31) the NIHSS explained ADL-dependence, but in that study the NIHSS was performed 2 days, and the BI 6 months post stroke. However, when it comes to cognitive functions, it is very important to be aware that NIHSS has limited diagnostic accuracy for identifying cognitive changes in acute stroke settings, especially in those with mild to moderate stroke (32). Thus, the MoCA could be assumed more feasible as a primary assessment tool to identify possible difficulties in ADL performance related to cognitive functions in patients with mild to moderate stroke.

A combination of the cognitive functions screened with the MoCA, neurological deficits, and patients' age were partly associated with ADL dependence. The results from the present study partially confirm a review study (8) where assessments that were performed several month post stroke, showed that patients with mild neurological deficits and younger age are more likely to be ADL independent. However, in that study cognitive function was not identified as a significant variable. In contrast, cognitive functions assessed with a different cognitive tool was associated with ADL dependence (6). Cognitive malfunctioning is known to be related to older age (33, 34). Thus, interaction of these two variables should not be underestimated while interpreting the results. Partial associations were also found between ADL dependence and motor variables of NIHSS - motor impairment in left upper extremity and right lower extremity. Studies showed that good motor function in the upper extremities (7, 8) could influence ADL performance. It is possible that more patients with relatively good functions in right upper extremity were included in the study, because MoCA assessment requires some motor skills, which is enough to hold a pen. Yet, it is still difficult to draw relevant conclusions about the results. As a conclusion, it could be assumed that a combination of stroke-related neurological severity, cognitive functions, and patients' age should be considered to explain ADL dependence at early stages of stroke onset.

To our knowledge this is the first and largest study where cognitive functions assessed with the MoCA were tested for ADL dependence at an early stage of stroke onset. However, there are several limitations and strengths of the study. There is a large proportion of incomplete data on the possible participants. This leads to a legitimate question about the validity of the study. The statistical analysis showed no significant difference regarding the sex between those with complete and incomplete data. Furthermore, there was age difference: more patients which higher age had incomplete data. It is possible that older patients had more severe stroke, thus assessments could not be performed within 36–48 h post stroke. Based on our data, we cannot do analyses and support this hypothesis. There is a risk that we have missed older patients. However, the study population of 550 patients had a wide range of age, the BI and the MoCA scores. Furthermore, in this study patients with mild to moderate stroke are included and there is no information about how cognitive functions assessed with the MoCA affects ADL outcomes in patients with severe stroke. However, the MoCA is a valid and reliable instrument for assessment of cognitive functions in patients with mild to moderate stroke (21). Thus, the study population could be assumed as representative. In addition, cognitive functions, and ADL performance were screened at the same time (36–48 h post stroke), leaving little room for changes in stroke-related symptoms between the assessments. The assessment instruments used in the study are valid and reliable in patients with stroke (4, 18). Thus, the study results probably could be generalized to the population of patients with mild to moderate stroke.

In conclusion, the results show that MoCA is associated with ADL and can partly explain ADL dependence 36–48 h post stroke. Patients' age and stroke-related neurological deficits should be taken in consideration for making more specific conclusions. More studies are warranted to understand if the MoCA can predict ADL dependence post stroke from a long- and short-term perspective. Identifying an optimal cutoff value of the MoCA could also be relevant for clinicians and patients for early planning of rehabilitation setting, management, and person-centered discharge from the hospital.

## Availability of data and material

According to the Swedish regulations https://www.epn.se/en/start/regulations/ the complete data cannot be made publicly available. Interested researchers may submit requests for data to the authors: contact professor Katharina S. Sunnerhagen, MD, Ph.D. E-mail: ks.sunnerhagen@neuro.gu.se.

## Author contributions

TA: acquisition of data, analysis, and interpretation of the data, drafting of the manuscript; LR: acquisition of data, design, and conceptualization of the study, revising the manuscript for intellectual content. ÅL-N: design and conceptualization of the study, revising the manuscript for intellectual content; KSS: design and conceptualization of the study, interpretation of the data, revising the manuscript for intellectual content.

### Conflict of interest statement

The authors declare that the research was conducted in the absence of any commercial or financial relationships that could be construed as a potential conflict of interest.
